# Exploration of the Chest in Health and Disease

**Published:** 1889-04

**Authors:** 


**Exploration of the Chest in Health and Disease.** By STEPHEN SMITH BURT, M. D., Professor Clinical Medicine and Physical Diagnosis in the New York Post-Graduate Medical School and Hospital; Physician to the Out-door Department (Diseases of the Heart and Lungs), Bellevue Hospital. Cloth, 12mo, pp. xiii., 206. New York: D. Appleton & Co. 1889.

This little volume presents a beautiful appearance. The printing, paper, and illustrations all add to its attractiveness. The latter especially deserve notice, and will be found most useful. Dr. Burt was induced to publish this manual by requests from his students for something embodying his methods of teaching. In the preface, he calls special attention to the importance of knowing the physiological anatomy of the heart and lungs, the relative position of the viscera to the parietes, and the physical signs that can be developed upon the normal chest, as the only true method of understanding the changes caused by disease. There are many works upon physical diagnosis, but we believe Dr. Burt's to be equal to any of them, and superior to most. Its contents are as follows: Introduction; Physical Methods of Diagnosis; Calormetation; Percussion; Auscultation; Diagnosis by Physical Signs of Diseases of the Lungs; Pneumonia; Pleurisy; Phthisis Pulmonalis; Exploration of the Heart; Heart-murmurs; Diagnosis by Physical Signs of Diseases of the Heart and of Thoraic Aneurism; and Aortic Stenosis. Every physician should possess this book.

